# On Rabies Canina

**Published:** 1806-11

**Authors:** Timothy Davis

**Affiliations:** Surgeon of His Majesty's Customs


### On Rabies Canina. By Timothy Davis, *Surgeon of His Majesty's Customs.*

THE minutest anatomical researches have not hitherto led to a discovery of the seat of rabies canina. A variety of conjectures has been made thereupon, and persons have permitted themselves a greater scope for presuming, from the circumstance of the trifling changes observable in parts parts supposed a priori to be the seat of the disease. Each has of course in his turn imagined that he had ascertained its real situation.

Certain phenomena that supervene during the action of this virus, lead us to conclude, its action is confined to the brain and nervous system, and every observer must have noticed that a greater or less degree of spasm seizes on the nerves, particularly when death approaches. Some persons are of opinion, that the bronchiæ are the seat of the disease, and assert, that the membrane which is expanded over them is generally in a state of inflammation. It is a fact that the voice of patients attacked with hydrophobia becomes extremely altered; sometimes it is very shrill, and at others exceedingly hoarse. Other physicians imagine the pharynx and the kidnies are the parts the first influenced by the virus; and the celebrated Mead thought it acted particularly on the vessels of the brain, producing plenitude there, and then throughout the whole sanguiferous system. The spinal marrow is by some considered the seat of rabies canina; but are there any remarkable appearances on dissection, that corroborate any one of these opinions, and refute the rest?

Diligent anatomists, who have examined the dead subject with a view to ascertain this point, have not, I believe, been so far demonstratively convinced, as to entirely decide upon so interesting an interrogatory. We may, however, observe here, that although marks of inflammation may be wanting in either of the parts alluded to, it is no proof that a disease, sui generis, cannot operate a change in the cerebral system, invisible indeed to us, but yet of so fatal a nature as promptly to put an end to life. Convulsive disorders proving fatal, frequently leave no marks of their seat behind them, though we do not hesitate to say, the cause of death is placed in the brain.

Passions of the mind and especially fear have been said to produce hydrophobia, and even to put an end to existence. I recollect being told of a case of spontaneous hydrophobia, in a young man that was amorous of a lady who refused listening to his offers of marriage. He bit his finger in a moment of despair, which a few days after became painful and considerably tumefied, next came on a horror of aliments, then of liquids of every kind, and he expired in convulsions.

Undoubtedly many instances of death from violent passion are on record, and fear has oftener than once been productive of fatal consequences to men as well as animals; mals; but I differ in opinion with some persons who think fear is commonly the cause of death in those that are bit by enraged dogs. As a proof that fear is not the principal agent in these cases, we may adduce the history of such infants as have been exposed to the bite of a mad dog, and who have also been victims to the virus. Nobody will say that fear was the cause of their death, but candidly allow it was attributable to something else.

However much fear may be assigned as an excitatory cause to determine the operation of the virus, it cannot therefore be looked upon as the source of it.

It is as difficult to define the nature of this virus, as that of small-pox, pox, and scrophula. We know it exists, that it has a particular march, and attacks, like them, certain parts in preference to others, but we are very ignorant of its properties. A great peculiarity is its not manifesting its effects in a given time, as some other kinds of virus do, though the state of the constitution, and especially of the moral may explain, as far as can be explained, the reason of the great difference there is in the periods of attack. In some persons the virus shall manifest its action in a few days, as in eight days or twenty; sometimes forty will elapse, three months, a year, and even a still longer period before it will be developed. It seems to lie dormant for a shorter or longer space of time, awaiting, as it were, a moment to be put in action, and in many cases persons never become enraged at all, notwithstanding it shall have been pretty well ascertained that the animal which inflicted the wound was really mad. I should however, be inclined to think, in the latter cases, that the virus had either not been introduced, or that it had been removed before absorption took place.

An important question to be resolved is this. Does the virus of hydrophobia remain latent and inactive in a wound, until a few days previous to the developement of the disease, or is it in all cases quickly absorbed, but retarded in its operation from particular causes influencing the habit? On the solution of this interrogatory would depend the successful treatment of persons bitten at a remote period; for if the presumption was in favor of the virus remaining in the wounded part till near the period its effects became visible, we should in all cases cauterize the spot as low down as it was probable impression had been made. Even in wounds completely healed, perhaps it would be adviseable to adopt this plan, and entirely expose the part that was within the reach of the animal's tooth. We should, I apprehend, be more likely to succeed, by our preventive means, if the remedy was resorted to before lancinating pains and uneasiness began to declare themselves about the cicatrix, as such symptoms rather indicate the passage of virus through the absorbents. I should then, in every suspicious case, be glad to seize the moment free from pain to make use of the preventive remedy, as the one the most favorable for attaining my end.

I believe excision or cauterization is the only remedy in which we can place implicit confidence for preventing this dreadful disease, notwithstanding the various nostrums that have in all ages, and in all countries, been extolled as true antidotes to the virus.

The caustic has not been so generally employed as its long known efficacy seems to recommend. It was a remedy the Greek physicians availed themselves of for preventing hydrophobia, and it would have been criminal in men that practised the healing art not to employ it. Inattention to its real efficacy, or an unseasonable use of it, are probably the reasons it has not been more generally resorted to; to which may be added, the milder methods cried up as specifics by the ablest men. Amongst those arcana were mercurial frictions, warm salt water baths, anodyne and antispasmodic electuaries and pills, vomits, and cathartics.

When the virus is in action, a difficulty of swallowing aliments soon comes on, preceded generally by a disgust to every kind of liquid, the eyes sometimes become fixed, then roll about and have a glaring aspect, the voice changes, tremors come on, the pulse is quick and rather hard, the mouth is filled with froth, at other times it is dry, the respiration becomes short and quick; palpitations of the heart next succeed, partial contractions of the muscles, delirium, syncope, and death.

With regard to the treatment of persons in this advanced state, our chief aim should be to lower the force of the vis vitæ and allay irritation. With a view to this, bleedings both topical and general are necessary, emollient and anodyne enemas, large doses of opium, and warm diluting liquors, if the person can be got to swallow. Opium might be tried in friction, and great care taken that the person does no injury to himself during a convulsion.

